# Supplementary material for: Causal Relationship between Aging and Anorexia Nervosa: A White-Matter-Microstructure-Mediated Mendelian Randomization Analysis
Source: Biomedicines. 2024 Aug 16;12(8):1874. doi: 10.3390/biomedicines12081874 (PMC11351342; doi:10.3390/biomedicines12081874)
Supplement: Supplementary file 1 [file biomedicines-12-01874-s001.zip › Supplementary Figure/Figure S8/Leaveoneout plot of MR analysis for TL to ubm-b-1708.pdf]

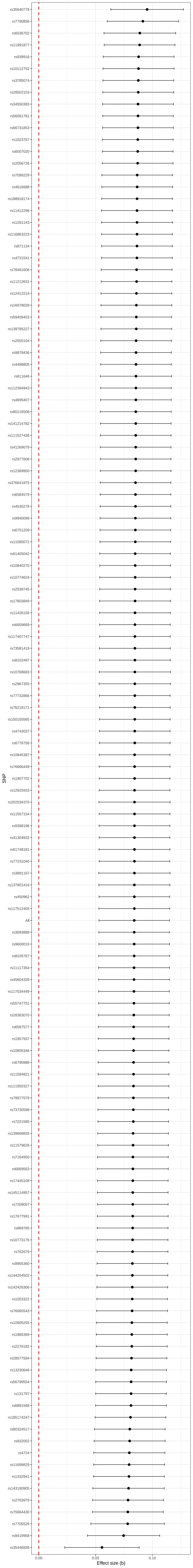
rs35640778
rs7790856
rs6536702
rs11991877
rs939916
rs10112752
rs3785074
rs28502153
rs34550383
rs56061761
rs66731853
rs1023767
rs6007020
rs2056726
rs7099229
rs4616688
rs188918174
rs11412296
rs1291143
rs116863223
rs871134
rs4731541
rs78491606
rs11212631
rs12412214
rs16978028
rs59409453
rs139795227
rs2555104
rs9878436
rs4498805
rs611646
rs112394943
rs4695407
rs80116508
rs141214782
rs111527438
rs41269079
rs2977608
rs12369950
rs376641875
rs6584579
rs4530278
rs9940099
rs6751209
rs11085072
rs61405042
rs10840270
rs10774624
rs2538745
rs17803849
rs11426156
rs6659669
rs117407747
rs73581419
rs8102497
rs10768683
rs2967355
rs77732866
rs76219171
rs150150565
rs4743037
rs6776756
rs10845387
rs76666449
rs1907702
rs12925933
rs202034370
rs11557154
rs9398196
rs41304832
rs61748181
rs77231040
rs3891167
rs137901416
rs450962
rs117512405
All
rs3093888
rs9600019
rs8105767
rs11117354
rs45604339
rs117034449
rs55747751
rs28363070
rs6587577
rs1957937
rs10805346
rs6790988
rs11584821
rs111950327
rs79977579
rs73730598
rs7221585
rs139669835
rs11579626
rs7164950
rs6669563
rs17445108
rs145114957
rs7209057
rs17677991
rs869785
rs10773176
rs762679
rs9955360
rs144204502
rs142426306
rs1003322
rs76065543
rs10905255
rs1985369
rs2276182
rs28577594
rs13230646
rs56799554
rs131797
rs6881568
rs185174247
rs80324517
rs932002
rs4724
rs11699829
rs1332941
rs143190905
rs2763979
rs75664430
rs7705526
rs9419958
rs35446936
SNP
0.00
0.05
0.10
Effect size (b)
